# Visualizing the multidimensional landscape of biological variation in modern microscopy

**DOI:** 10.3389/fbinf.2026.1757489

**Published:** 2026-03-10

**Authors:** Gesine F. Müller, Torben Göpel, Nico Scherf, Jan Huisken

**Affiliations:** 1 Multiscale Biology, Faculty of Biology and Psychology, Georg-August-University Göttingen, Göttingen, Germany; 2 Methods and Development Group Neural Data Science and Statistical Computing, Max Planck Institute for Human Cognitive and Brain Sciences, Leipzig, Germany; 3 Center for Scalable Data Analytics and Artificial Intelligence (ScaDS.AI), Leipzig, Germany; 4 Cluster of Excellence ‘Multiscale Bioimaging: from Molecular Machines to Networks of Excitable Cells’ (MBExC), Georg-August-University Göttingen, Göttingen, Germany; 5 Morgridge Institute for Research, Madison, WI, United States

**Keywords:** biological variation, light sheet microscopy, smart microscopy, representative sampling, visualization

## Abstract

Variation is a foundational biological principle that has historically been marginalized—both due to limited experimental accessibility and because of idealized, stereotypic blueprints rooted in essentialist thinking. With the advent of genetics and quantitative biology investigating environmental influences on the phenotype, variation was redefined from mere noise to a fundamental property. Modern light sheet microscopy now enables high-resolution, long-term imaging of dynamic processes across large populations, making it possible to systematically study phenotypic variation *in vivo*. Yet, the resulting high-dimensional datasets overwhelm traditional modes of analysis and visualization, risking the loss of biological insight. The transition from qualitative representation to quantitative measurement demands new epistemic practices—shifting from selective human interpretation to computational abstraction. Instead of relying on either very limited sampling or exhaustive scanning, we advocate for representative sampling of phenotypic variation: adaptive, model-guided systems that dynamically sample biological variation using real-time feedback, directing attention towards biologically relevant events and rare or extreme phenotypes. The underlying models act as the interface to human insight, constructing navigable, queryable representations of variation as a multidimensional manifold shaped by genetics, environment, and stochasticity. Crucially, adaptive systems call for new methods of visualizations—interfaces that encode uncertainty, consensus, and distributional structure. Such visualizations should preserve the interpretability of historical illustrations while fully embracing biological variation. The future of biology lies not in acquiring more data, but in developing smarter ways to sample, represent, and understand it.

## 1 Variation is a foundational biological principle

Biological variability is a central topic in modern biology. Equally central are the ways in which this phenomenon can be studied and represented. In this perspective, we argue that advances in data acquisition demand corresponding changes in visualization practices to adequately capture and reason about biological variation, and that these demands have fundamentally shifted the role of visualization in the scientific intellectual process.

Historically, variation in biological traits and processes has often been overlooked due to limited experimental accessibility as well as idealized, stereotypic thinking. From Aristotle’s essential forms in *De Generatione Animalium* (Aristotle, 1943) to Goethe’s archetype, *Urpflanze, Urthier*, (von Goethe, 1817), biological forms were perceived as manifestations based on static blueprints. Goethe’s search for an archetype aimed to find a universal structure behind individual differences, since “the particular can never be representative of the whole” (von Goethe, 1817; own translation), and the hidden types were considered truer to nature and more real than the individual specimen (Vogt et al., 2022). This effort to extract the typical from the variable continued into modern science, where idealistic representations were used as a means to achieve objectivity (Daston and Galison, 1992). Replacing metaphysical archetypes with mathematical and geometric laws, D’Arcy Thompson’s *On Growth and Form* (Thompson, 1917) still followed this blueprint logic, seeking universal order behind biological diversity. Such idealized concepts have long shaped biological research on form and function, leaving no room for variations in biological processes—the prevailing paradigm was only to be challenged with the advent of genetics.

With Mendel’s *Experiments on Plant Hybridization* (Mendel, 1866), the understanding of trait inheritance shifted from a random to a systematic process. Later, work on chromosomal mechanisms and gene interactions extended this understanding of heredity beyond simple ratios (Morgan et al., 1915). Genetics thus became one of the first scientific fields dedicated to studying variability itself. At the same time, the rise of biological statistics (Fisher, 1918; Fisher and Mackenzie, 1923), highlighted the influence of environmental factors on the formation of phenotypes and laid the foundation for the concept of phenotypic plasticity (West-Eberhard, 1989). Extending this shift into developmental biology, Waddington’s epigenetic landscape metaphor conceptualized development as a probabilistic process resulting in distributions of developmental outcomes (Waddington, 1956; Waddington, 1957). Together, these developments in genetics as well as quantitative work of environmental influences on the phenotype not only acknowledged variations in biological processes but redefined it—from being seen as mere noise to being understood as a fundamental principle of life (Monod, 1974).

Variation in phenotypic traits among individuals arises from genetic differences and two non-genetic sources, namely, environmental influences and intrinsic stochasticity both driven by epigenetic mechanisms. In particular stochastic fluctuations involved in cellular and molecular processes such as gene expression generate variability even among genetically identical individuals in identical environments. The concept of reaction norms captures how a single genotype can produce a range of phenotypes under varying conditions, representing a reservoir of adaptive potential (Vogt, 2020).

## 2 From representation to quantification: the historical turn

Biological structures and developmental processes have long been studied through direct observation, relying primarily on the human eye and aided by early optical instruments. In the past, these observations were documented manually through drawings, which served as an empirical record and conceptual interpretation. Scientists acted as both selective and interpretive agents in their work—choosing representative samples, filtering observations, and deciding what to include in their cumulated drawings (Sulston et al., 1983). Representation was thus already a highly synthetic form of integration: each image condensed multiple observations into a coherent visual consensus. This approach to scientific representation arises from the naturally evolved cognitive processes of abstraction and categorization of visually perceived objects (Daelli et al., 2010). However, these processes and thus ultimately representation of objects, their features, and putative similarities are significantly shaped by background knowledge and experience (Tanaka et al., 2012). Therefore, the traditional means of representation of biological traits have been laden with subjectivity arising from the interpreter’s experience.

Microscopy, long a cornerstone of biological research, has undergone a profound transformation from mere enhancement of the human eye into a data-driven and quantitative discipline. Fast-forwarding to modern microscopy, the rise of digital imaging (Morrison and Gardner, 2015) and computational analysis has turned representation into measurement (Culley et al., 2024; Woodhams and Uhlmann, 2025). Biological research has benefited greatly from advances in machine learning, particular deep neural networks, transformer architectures, and foundation models adapted to microscopy modalities, such as µSAM (Archit et al., 2025; Hein et al., 2025; Cao et al., 2025). These methods support among others segmentation, object detection, and representation learning to quantify morphological variation at scale (Tsutsumi et al., 2023; Archit et al., 2025). Deep learning frameworks have been integrated into existing microscopy workflows, facilitating reproducible and scalable image analysis (Von Chamier et al., 2021; Gómez-de Mariscal et al., 2021; Bragantini et al., 2025; Huijben et al., 2025). Beyond image quantification, machine learning techniques are increasingly used to investigate biological variation across scales, including genotype-to-phenotype relationships and inter-individual variability: Deep generative models, particularly variational autoencoders, learn latent trait representations from high-dimensional data and enable simulation of phenotypic diversity in, e.g., single-cell transcriptomics (Treppner et al., 2022; Seninge et al., 2021). Graph neural networks and explainable AI further support interpretable modeling of structured biological systems revealing subtle inter-individual differences (Kawano, 2025; Godesberg et al., 2024). Collectively, these machine learning methods enable the integration of large and heterogeneous datasets, opening new avenues for studying phenotypic variation and its driving factors.

A particularly transformative development has been light sheet fluorescence microscopy, which enables fast, gentle, and high-resolution imaging across the spatial and temporal dimensions of biological processes (Huisken et al., 2004; Power and Huisken, 2017). By projecting a thin sheet of light into the sample and collecting the emitted fluorescence orthogonally, optical sectioning is achieved with minimal photodamage. This technique provides unprecedented possibilities to capture biological variation as it unfolds *in vivo*—revealing not just representative forms, but entire distributions of developmental trajectories for large numbers of samples (Shah et al., 2019). Furthermore, light sheet microscopy addresses many intrinsic experimental constraints—balancing sample health, spatial and temporal resolution, and signal-to-noise ratio (Morgado et al., 2024).

This shift from qualitative representation to quantitative measurement in microscopy has introduced new challenges. High-dimensional datasets are not readily interpretable, but require computational methods to transform them into meaningful representations. If phenotypic variation is studied by multi-sample imaging, the scale of the datasets becomes even harder to process. While modern microscopy now produces data of unprecedented richness, the very scale and complexity of these datasets may obscure, rather than clarify, biological variation. The historical turn from representation to quantification marks not only a technological transformation but also a shift in epistemic practice: from interpretive synthesis to computational abstraction, and from (selective) seeing to recording. Cognitive integration—historically achieved through selective observation and interpretation—has been displaced by computational integration, demanding new conceptual frameworks to bridge data and understanding.

## 3 Case study: multi-sample neuronal activity in zebrafish development

While variation in shape is already challenging to describe, variation in dynamic traits is even more difficult to represent and interpret. Brain activity is one striking example that highlights this complexity: how do patterns of coordinated activity across neurons emerge and organize during development. How do functional assemblies establish themselves as the nervous system matures? The interplay between genetic makeup and functional wiring underlies this dynamic process, shaping connectivity patterns and coordinated activity (Blankenship and Feller, 2010; Barabási et al., 2024).

**FIGURE 1:**
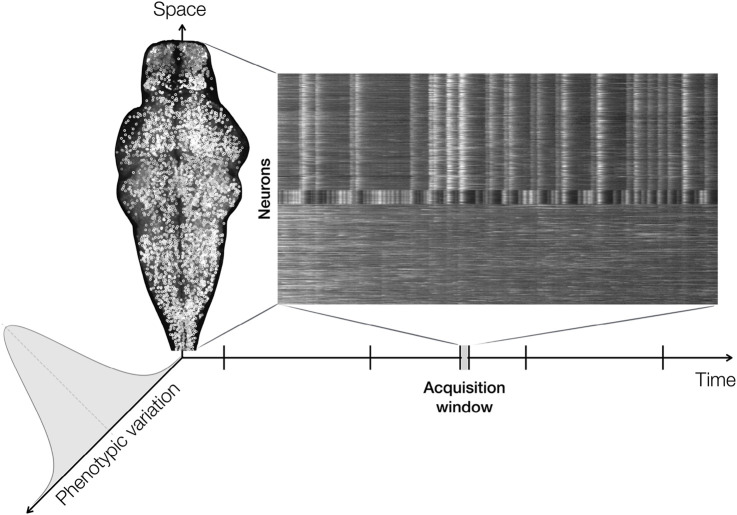
Dimensions of neuronal activity during development. Neuronal activity evolves across the developmental time domain of zebrafish larvae. Calcium traces are acquired within a small time window that is swept along the developmental axis. Single nuclei are segmented, and their normalized and sorted calcium traces are displayed as a stacked kymograph with neurons on the y-axis and acquisition time on the x-axis. Developmental time windows are additionally stacked with a high sample size to capture the variation of neuronal activity development.

Using zebrafish larvae as a model system, this process can be followed continuously making use of transgenic lines expressing a genetically encoded calcium indicator fused to a fluorescent protein in nearly every neuron (Park et al., 2000). When combined with light sheet microscopy, this approach enables long-term imaging of the whole brain at high spatial and temporal resolution across multiple individuals—thus directly capturing the biological variation development of neural activity (Figure 1). However, processing these data simply by *looking through them* far exceeds our cognitive capacity. The raw data therefore require processing that is targeted and validated for the respective scientific question: In this example, machine-driven image analysis needs to extract the meaningful calcium traces corresponding to active neurons. Only after the meaningful data have been distilled from the overwhelming raw input, attempts can be made to make the data accessible to the researcher for further interpretation, visualization, and communication. A representation that provides a rapid visual impression of the brain-wide dynamics within a single sample is a stacked kymograph of the extracted fluorescent traces from active neurons. Yet, most subsequent analysis methods—whether traditional or machine learning–based—remain primarily optimized for single-sample datasets. The key challenge lies in extending these methods to incorporate multi-sample variation already at the stage of data integration.

Without appropriate abstraction and representation, especially of the multi-sample dimension, the richness of imaging data can obscure rather than elucidate the organizing principles of neuronal activity. The cognitive load increases as data volume grows, and biological meaning is at risk of being lost in computational complexity. Ultimately, biological understanding does not emerge directly from raw data, but from how variation is represented, contextualized, and interpreted. The open question remains: How can we represent and visualize a complex, highly variant process such as the developmental emergence of neuronal activity—explicitly encoding biological variation rather than reducing it away?

## 4 Seeing variation, seeing biology

Many sophisticated analysis pipelines still operate on single samples or at best can compare a small number of them. Even recent integrative frameworks rarely attempt to explore the full distribution of biological variation (Schneider et al., 2023; Schneider et al., 2025). However, a distribution of biological variation is not a discrete and well-delineated structure but can be represented as a manifold shaped by genetics, environmental influences and stochasticity—every sample represents a certain phenotype in this multidimensional space of traits. Our entry point to this distribution is often by means of microscopy. But how to efficiently capture and understand the nature of this distribution? To illustrate possible approaches, we sketch two opposing scenarios: the classical and the exhaustive acquisition.

**FIGURE 2:**
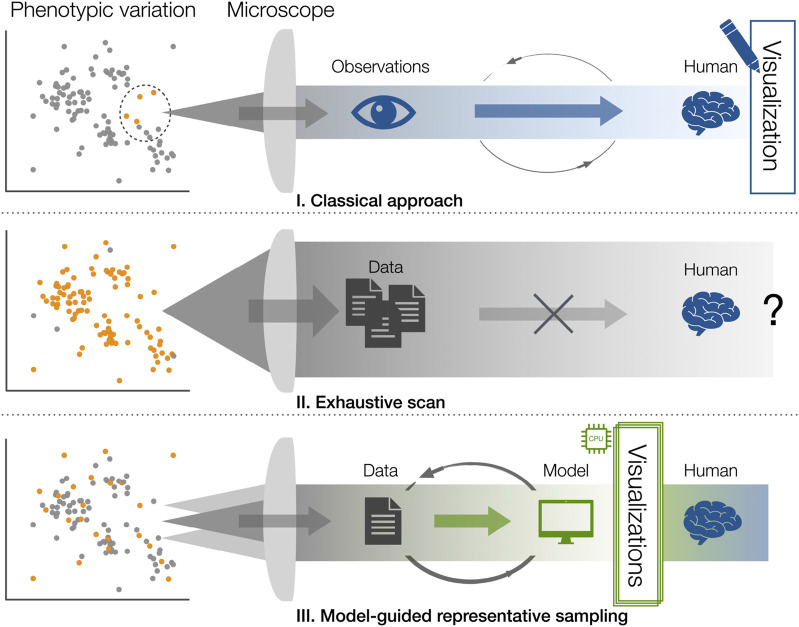
Conceptual workflows of sampling biological variation. A subset of a population exhibiting phenotypic variation is sampled (orange) and made accessible with different strategies: **(I)** Classical approach: sparse, experiment-limited sampling of a larger biological distribution; data acquisition and interpretation proceed sequentially and depend heavily on the scientist’s selection and intuition. **(II)** Exhaustive scan: an attempt to sample the full distribution, generating overwhelming redundancy and unprocessable amounts of data. **(III)** Model-guided representative sampling: a feedback-driven strategy in which a microscope and explicit model jointly explore the distribution, selectively acquiring informative data while building a navigable representation of biological variation.

In traditional experimental workflows (Figure 2I), we sample the distribution sparsely and somewhat arbitrarily. While not truly random, the samples we collect are constrained to what we can feasibly access experimentally. The microscope is used to capture these observations, they are processed and sorted, and a human, the scientist, interprets them to answer a biological question. The results are distilled into a visualization—historically even hand-drawn—for interpretation, communication, and ultimately publication. While there is some iterative refinement between scientist and microscope, the process remains fundamentally linear and selective at its core. We glimpse at the distribution only through a narrow keyhole. An opposing extreme sampling case might be capturing everything—a full, multi-dimensional dataset from every accessible sample in the population (Figure 2II). But this quickly becomes unmanageable: terabytes of redundant data from nearly identical specimens, no tractable way to process or analyze it, and no plausible route towards visualizing the outcome. Such a fully exhaustive strategy is neither feasible nor desired. Excessive data accumulation does not equate to biological understanding.

Instead of blindly or exhaustively collecting data, we aim to acquire representative datasets—guided by a model that is coupled directly to the microscope (Figure 2III). The microscope still captures the raw data, but a tight feedback loop dynamically determines when, where and how to image next. This explicit model can take many forms: from a simple process model encoding biological expectations to a sophisticated reinforcement-learning agent exploring and representing the sample space (Milosevic et al., 2024). The model serves dual purposes: directing the acquisition by interacting with a robot-like microscope and constructing an intermediate representation using the incoming data stream—a statistical model that progressively aligns with the manifold of the underlying biological variation and can be both queried or used for visualization purposes. The scientist no longer manually steers data acquisition, hand-picking datasets and preparing the processing. Instead, they interact primarily with the model-driven visualizations—representations that encode variation instead of reducing it away or being hidden in the sheer amount of data. As the sample size grows, data flow becomes more manageable, because highly redundant observations are avoided as long as the distribution is sampled sufficiently to estimate rates. This strategy also allows targeted investigation of rare or extreme cases—the edges of the distribution—which often prove to be particularly valuable to further understanding. Ultimately, visualization becomes the essential interface between complex biological variation and the human mind. Understanding biological processes emerges only once variation is made tangible.

## 5 Smart microscopy to the aid?

The technical realization of a truly representative sampling is key to an objective acquisition of biological variation. After providing the basic rationale, such a model needs to reliably interpret its own observations regarding their relevance without the necessity of continuous feedback from the researcher. The researcher, on the other hand, needs to have confidence in the performance of the model and the representativeness of the acquired data. Smart microscopy—also referred to as adaptive or feedback-driven microscopy—describes, in its basic form, imaging systems that dynamically adjust their acquisition strategy in response to the sample, imaging conditions, or experimental objectives. Rather than relying on fixed, manually defined parameters, these systems incorporate real-time adaptations throughout the workflow to minimize photo-damage and data redundancy (Scherf and Huisken, 2015; He and Huisken, 2020; Mahecic et al., 2022; Daetwyler and Fiolka, 2023; Hinderling et al., 2026). Automated control frameworks further handle experimental logistics such as sample localization, auto-focus, and field-of-view correction (Royer et al., 2016; Marin et al., 2024). Central to all of these efforts is to close the loop: acquisition, data, acquisition—where analysis directly informs the next experimental step (Carpenter et al., 2023). Viewed together, these developments mark a shift from automation toward collaboration: the microscope evolving from a passive recorder into an active scientific partner (Kesavan and Nordenfelt, 2026). More narrowly defined approaches such as compressed sensing, active learning, and adaptive sampling address specific aspects of data-efficient image acquisition and can be viewed as constituent strategies within a smart microscopy framework. Compressed sensing exploits prior information to reconstruct under-sampled measurements, often in conjunction with masks or structured illumination (Calisesi et al., 2022). While highly effective for accelerating acquisition or reducing data volume, compressed sensing does not intrinsically guide decision-making during an ongoing experiment. Active learning focuses on optimizing annotation effort in supervised learning by identifying informative samples for labeling by an expert (Cao et al., 2025; Bilodeau et al., 2024). In microscopy, this has proven valuable for segmentation and data-centric workflows, yet the adaptivity primarily concerns what is labeled rather than how measurements are acquired. Adaptive sampling methods operate at the acquisition level, dynamically adjusting how densely data are collected based on prior observations or uncertainty estimates, and alternatively in post by adaptively representing data richness (Cheeseman et al., 2018; Ye et al., 2025).

Modern microscopy faces a dilemma: as measurements become more precise and high-dimensional, intuitive understanding of biological variation becomes buried under its own abundance. The integration of model-guided representation sampling and visualization as an interpretative interface between computation and humans addresses this challenge. The intermediate model functions as an active agent that learns relevant representations of the biological process and steers data acquisition accordingly. By prioritizing relevance over raw throughput, the information-to-data ratio is improved reducing the total volume of acquired data while increasing its biologically meaningful proportion. The concept of smart microscopes with integrated visualizations might unlock the next level towards the true incorporation of biological variation in science.

## 6 From adaptive acquisition to human insight: a change in the epistemological process

The promise of smart microscopy paired with an visualization interface is that the microscope becomes an active collaborator: it decides what, when, and where to sample from a distribution. Yet, while a powerful concept, its implementation introduces two profound challenges—representativeness and interpretability. Model-guided representative sampling fundamentally changes the acquisition process by relying on models that estimate or learn phenotypic distributions. A model’s bias towards already explored states or towards *a priori* assumptions requires careful balancing of exploration and exploitation to ensure that adaptive acquisition remains grounded in the true underlying distribution. Especially as model complexity increases, representative sampling inherits well-known problems from active learning, including sampling bias and sensitivity to model hyperparameters (Bengio, 2009; Sinz et al., 2019). These issues are exacerbated as model complexity increases and as feedback loops between prediction and acquisition tighten. Practical constraints further limit what model-guided acquisition can achieve. Imaging speed, phototoxicity, and photobleaching restrict how frequently a system can adapt its sampling strategy. Computational latency and resource demands may also limit real-time decision-making, forcing compromises between model sophistication and experimental throughput. Complex models that require extensive training to perform adequately may rely on offline pre-training on simulated data (Bilodeau et al., 2024) and a suitable computational infrastructure.

A second challenge is creating visualizations that are meaningful and interpretable for the human mind. Historical illustrations—made *from* the human mind *for* the human mind—integrated many observations into an immediately interpretable form. These forms of visualization, however, increasingly fall short in modern microscopy that targets dynamic processes and their inherent variation. A natural response might be to turn to dynamic, interactive, or even fully immersive ways of data exploration (Skarbez et al., 2019). Yet, such complex visual environments often shift the burden of interpretation onto the viewer, who must learn to navigate an unfamiliar space. Presenting more data in these settings does not necessarily provide a better overview or a clearer understanding, but relocates the problem to the user. Approaches from other domains, such as uncertainty-aware visualization, offer partial inspiration, but they do not fully resolve the challenge of representing high-dimensional, dynamic biological variation (Sterzik et al., 2025).

What we now need in modern microscopy—generating large and multidimensional data on biological variation—are visual strategies that recover the descriptive power of historical illustration while explicitly encoding variation: visualizations that compare subclasses within a distribution, encode redundancy or consensus strength, and reveal how processes diverge or cluster over time. These visualizations serve as the interface between model-driven decisions and biological interpretation—marking a change in their role in the epistemological process: from being a mere product of the researcher’s interpretation towards being the gateway for initial understanding. However, if uncertainty, variability, or model confidence are not clearly conveyed, visualization outputs may appear precise while masking their underlying assumptions. Smart microscopes will require equally smart visualization to make data acquisition transparent and biological variation intelligible. As biology embraces variation as foundational, we must reinvent the tools that let us see it.

## Data Availability

The raw data supporting the conclusions of this article will be made available by the authors, without undue reservation.
